# Distinct Trajectories of Consciousness Recovery During Targeted Temperature Management in Out-of-Hospital Cardiac Arrest Survivors: A Cluster Analysis

**DOI:** 10.3390/medicina62030427

**Published:** 2026-02-24

**Authors:** Hyo Joon Kim, Sang Hoon Oh, Kyu Nam Park, Jee Yong Lim

**Affiliations:** 1 Department of Emergency Medicine, Seoul St. Mary’s Hospital, The Catholic University of Korea, Seoul 06591, Republic of Korea; 2 International Healthcare Center, Seoul St. Mary’s Hospital, The Catholic University of Korea, Seoul 06591, Republic of Korea

**Keywords:** late awakeners, recovery trajectory, prognostication

## Abstract

*Background and Objectives*: Static prognostication in comatose out-of-hospital cardiac arrest (OHCA) survivors may overlook delayed recovery, risking premature withdrawal of life-sustaining therapy (WLST). This study aimed to identify distinct longitudinal phenotypes of consciousness recovery and determine the prevalence and characteristics of the Late Awakener phenotype. *Materials and Methods*: We applied K-means clustering to serial Glasgow Coma Scale motor scores (0, 24, 48, 72 h, Day 5) in 417 adult OHCA survivors treated with targeted temperature management at Seoul St. Mary’s Hospital (2009–2023). *Results*: Three distinct phenotypes emerged: Early Awakeners (*n* = 86, 20.6%), Late Awakeners (*n* = 54, 12.9%), and Non-Awakeners (*n* = 277, 66.4%). While Early Awakeners had 96.5% good neurological outcomes at 6 months, 79.6% of Late Awakeners also achieved good outcomes despite being indistinguishable from Non-Awakeners at 48 h (mean GCS motor score ≤ 2). Late Awakeners had significantly higher rates of shockable rhythms (72.2% vs. 21.3%, *p* < 0.001) compared to Non-Awakeners. *Conclusions*: The identification of a Late Awakener phenotype—comprising 13% of the cohort and one-third of all survivors with good outcomes—challenges early prognostic pessimism. An extended observation window of at least 5–7 days may be warranted for patients with shockable rhythms to avoid premature WLST, even when early motor responses are absent.

## 1. Introduction

Out-of-hospital cardiac arrest (OHCA) remains a leading cause of mortality and severe neurological morbidity worldwide, with survival rates hovering around 10% despite advances in resuscitation science and post-cardiac arrest care [1,2]. Among those who achieve return of spontaneous circulation (ROSC), hypoxic-ischemic brain injury (HIBI) is the primary determinant of poor outcome and the leading cause of death, often accounting for two-thirds of mortality in patients who survive to hospital admission [3,4]. Consequently, accurate neuroprognostication has become a cornerstone of post-resuscitation care, serving as the critical basis for decisions regarding the maintenance or withdrawal of life-sustaining therapy (WLST) [5].

Current international guidelines, such as those promulgated by the European Resuscitation Council (ERC) and the European Society of Intensive Care Medicine (ESICM), advocate for a multimodal prognostication algorithm delayed until at least 72 h post ROSC to allow for the clearance of sedative effects and potential neurological recovery [5]. Within this framework, the clinical examination remains the most fundamental and accessible tool. Specifically, the Glasgow Coma Scale (GCS) motor score is heavily weighted [6]; a motor score of M1 (no response) or M2 (extension) at 72 h, particularly when combined with other malignant markers such as absent pupillary light reflexes or elevated neuron-specific enolase (NSE), is widely accepted as a robust predictor of poor neurological outcome [7,8].

However, a critical and often overlooked limitation of these conventional algorithms is their reliance on static snapshots of neurological status assessed at fixed time points (e.g., 24, 48, or 72 h). This binary approach classifying a patient as either awake or comatose at a specific hour fails to capture the complex temporal dynamics of brain recovery [9]. Emerging evidence suggests that neurological recovery after global ischemia is not a uniform event but a heterogeneous longitudinal process with varying velocities and trajectories [10,11]. The awakening process can be significantly modulated by individual factors such as the extent of initial injury, the efficacy of cerebral reperfusion, and the clearance rate of sedative agents used during targeted temperature management (TTM) [12].

Of particular concern is a subset of patients who may be characterized as Late Awakeners. These individuals exhibit prolonged unresponsiveness beyond the standard 72-h observation window often indistinguishable from those with irreversible brain injury yet eventually achieve favorable functional outcomes [13,14]. The underlying mechanisms for this delayed recovery may involve reversible phenomena such as stunned brain tissue, prolonged metabolic depression, or delayed synaptic reorganization, rather than permanent neuronal death [15]. In such cases, strict adherence to early prognostic cut-offs creates a significant risk of a self-fulfilling prophecy, where potentially recoverable patients are subjected to premature WLST based on an initial lack of motor response [16,17]. While recent studies have emphasized the danger of early prognostication and the grey zone of indeterminate outcome [18,19], few have systematically characterized the longitudinal patterns of consciousness recovery using granular, time-series data.

This gap highlights the need for a shift from static prediction to dynamic phenotyping. Just as sepsis and ARDS are now understood as heterogeneous syndromes requiring precision phenotyping, post-cardiac arrest coma may also comprise distinct recovery classes that require tailored management strategies [20,21]. Identifying specific trajectories of motor recovery could allow clinicians to distinguish between patients with irreversible injury and those with delayed but viable recovery potential who require an extended observation window.

Therefore, this study aimed to move beyond static prediction models. Using a data-driven cluster analysis of serial GCS motor scores collected from admission to Day 5, we sought to: (1) identify distinct longitudinal phenotypes of consciousness recovery in a cohort of comatose OHCA survivors treated with TTM; (2) determine the prevalence and characteristics of the Late Awakeners phenotype; and (3) evaluate whether these specific delayed recovery trajectories are associated with favorable long-term neurological outcomes, thereby challenging the conventional pessimistic interpretation of early poor motor responses.

## 2. Materials and Methods

### 2.1. Study Population

This retrospective cohort study included patients admitted to Seoul St. Mary’s Hospital between January 2009 and December 2023. We screened all adult patients (≥18 years) with non-traumatic OHCA who achieved sustained ROSC (≥20 min) and were treated with targeted temperature management (TTM). Exclusion criteria were: (1) traumatic etiology of arrest; (2) known pre-existing severe neurological disability (Cerebral Performance Category [CPC] 3 or 4); (3) Do-Not-Attempt-Resuscitation (DNAR) orders established before ROSC; (4) death within 24 h of admission, precluding longitudinal assessment; and (5) incomplete data on serial GCS assessments.

### 2.2. Post-Cardiac Arrest Care

Post-cardiac arrest care was provided in accordance with our institutional protocol based on the international guidelines relevant to each period [22,23]. TTM was induced using surface (Arctic Sun^®^; BD, Franklin Lakes, NJ, USA) or intravascular (Thermogard XP^®^; ZOLL, Chelmsford, MA, USA) cooling devices. The target temperature was maintained at 33 °C or 36 °C for 24 h [24], followed by controlled rewarming (0.25 °C/h). Sedation and analgesia were maintained with midazolam/remifentanil infusions. Neuromuscular blocking agents (NMBAs) were administered during the induction of TTM to prevent shivering but were discontinued or minimized during the maintenance and rewarming phases to facilitate neurological examination, unless strictly indicated for shivering control or ventilator asynchrony.

### 2.3. Data Collection and Outcome Definition

The primary variable of interest was the GCS motor score (GCS-M), assessed at five standardized time points: admission (0 h), 24 h, 48 h, 72 h, and Day 5 post-ROSC. All neurological examinations were performed by trained critical care nurses or physicians. To minimize the confounding effect of sedation, GCS scores were recorded after interrupting sedation (sedation vacation) whenever clinically feasible, or at the nadir of sedation depth. Sedation vacations were performed daily during the maintenance and rewarming phases of TTM. Sedative infusions (midazolam and remifentanil) were controlled to the minimum dose required to maintain patient safety. Neurological assessments were conducted after a minimum of 30 min off sedation, or at the nadir of sedation depth when complete discontinuation was not clinically feasible due to concerns such as ventilator asynchrony or hemodynamic instability. The primary endpoint was the neurological outcome at 6 months after cardiac arrest, assessed using the CPC scale [25]. A good neurological outcome was defined as CPC 1 (good cerebral performance) or 2 (moderate disability), while a poor outcome was defined as CPC 3–5.

### 2.4. Statistical Analysis: Trajectory Modeling

To identify distinct longitudinal phenotypes of consciousness recovery, we utilized K-means clustering, an unsupervised machine learning algorithm. We constructed a vector of serial GCS-M scores (M_0_, M_24_, M_48_, M_72_, M_Day5_) for each patient. Missing values in the GCS trajectory were handled using the Last Observation Carried Forward (LOCF) method for survivors to reflect the clinical reality that neurological status tends to plateau or improve, while patients who died (CPC 5) were assigned a GCS-M score of 1 for all subsequent time points. To ensure the stability of this imputation strategy, we performed a sensitivity analysis excluding patients who died before Day 5; this analysis confirmed that the trajectory structures remained consistent, although the specific results are not presented in this article. The optimal number of clusters (k) was determined based on the Elbow method and clinical interpretability, which suggested three distinct phenotypes. Continuous variables were assessed for normality using the Shapiro-Wilk test. Given the non-normal distribution of key variables, continuous data are presented as medians (interquartile ranges [IQR]) and were compared using the Kruskal-Wallis test, followed by Dunn’s post-hoc test for pairwise comparisons. Categorical variables are presented as frequencies (%) and were analyzed using the Chi-square test or Fisher’s exact test, as appropriate. All statistical analyses were performed using Python (version 3.9) and R software (version 4.2.0). A two-sided *p*-value of <0.05 was considered statistically significant.

## 3. Results

### 3.1. Study Population and Baseline Characteristics

During the study period, a total of 465 patients were screened. After excluding patients who died within 24 h of admission or had incomplete longitudinal data, 417 patients were included in the final analysis. The median age of the cohort was 56.0 years (IQR 44.7–69.0), and 71.5% (298/417) were male. A shockable rhythm was present in 38.8% of patients, and 67.1% had a witnessed arrest. The median time from arrest to ROSC was 31.0 min (IQR 18.0–42.8).

Detailed baseline characteristics stratified by the identified recovery phenotypes are summarized in Table 1.

### 3.2. Identification of Longitudinal Recovery Phenotypes

**Figure 1:**
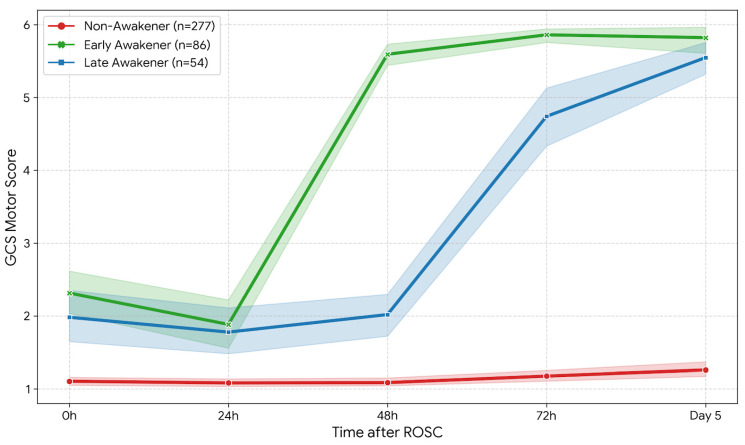
Longitudinal trajectories of consciousness recovery based on serial GCS motor scores. The lines represent the mean Glasgow Coma Scale motor (GCS-M) scores for each phenotype over the first 5 days post-arrest. Shaded regions indicate 95% confidence intervals. Green line: Early Awakeners (*n* = 86), showing rapid improvement to near-full responsiveness within 48 h. Blue line: Late Awakeners (*n* = 54), characterized by delayed awakening; while initially indistinguishable from Non-Awakeners, they show a surge in recovery starting after 48 h. Red line: Non-Awakeners (*n* = 277), exhibiting persistent coma (GCS-M ≤ 2) throughout the observation period.

The K-means clustering algorithm, applied to serial GCS motor scores from admission to Day 5, identified three distinct longitudinal phenotypes of consciousness recovery (Figure 1). Early Awakeners (*n* = 86, 20.6%): This group exhibited rapid neurological recovery. Their mean GCS-M score improved from 2.3 at admission to 5.6 within 48 h. By Day 5, nearly all patients in this group had regained full motor responsiveness. Late Awakeners (*n* = 54, 12.9%): This phenotype represented a distinct subgroup of patients characterized by delayed awakening. Crucially, their motor scores during the first 48 h (mean GCS-M ≤ 2.0) were statistically indistinguishable from those of the Non-Awakeners. However, unlike the non-awakeners, this group demonstrated a rapid surge in consciousness commencing after 48 h, achieving a mean GCS-M score of 4.7 at 72 h and further improving to 5.6 by Day 5. Non-Awakeners (*n* = 277, 66.4%): This group comprised the majority of the cohort and showed persistently poor motor responses (GCS-M 1–2) throughout the entire 5-day observation period, reflecting severe and irreversible brain injury.

### 3.3. Association Between Recovery Trajectories and Long-Term Outcomes

**Figure 2:**
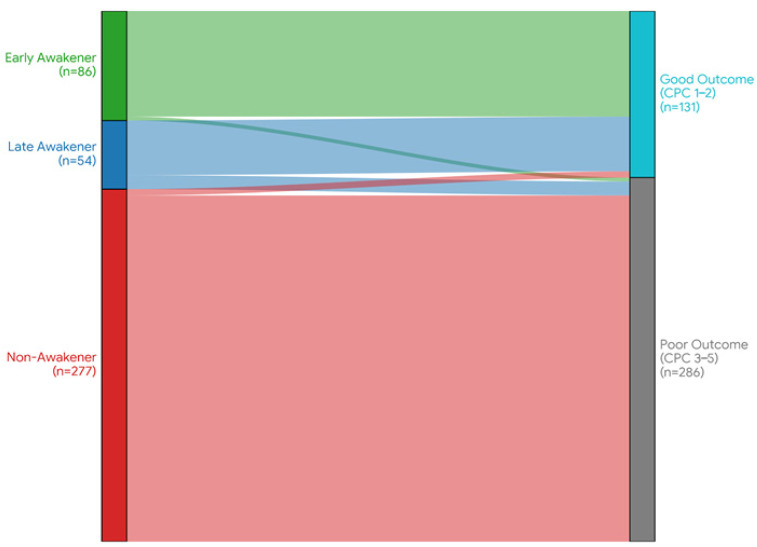
Alluvial plot illustrating the prognostic flow from recovery phenotypes to 6-month neurological outcomes. The diagram visualizes the transition of patients from their identified recovery phenotype (left axis) to their final neurological outcome (right axis: Good Outcome [CPC 1–2] vs. Poor Outcome [CPC 3–5]). The height of each stream is proportional to the number of patients. Early Awakeners (*n* = 86, green) are strongly linked to Good Outcome. Non-Awakeners (*n* = 277, red) predominantly progress to Poor Outcome. Notably, the Late Awakeners (*n* = 54, deep blue)—represented by the blue stream—demonstrate a substantial flow toward Good Outcome (79.6%), highlighting the potential for delayed recovery despite initial poor motor scores.

The primary outcome of good neurological recovery (CPC 1–2) at 6 months varied significantly across phenotypes. As illustrated in the alluvial plot (Figure 2), the flow of patients from recovery phenotypes to final outcomes demonstrates a clear divergence. Early Awakeners achieved a near-universal good outcome (96.5%, 83/86). Conversely, Non-Awakeners had a dismal prognosis, with only 1.8% (5/277) achieving CPC 1–2. Most notably, the Late Awakeners—despite their initial profound coma—achieved a good neurological outcome in 79.6% (43/54) of cases. This rate was not significantly different from that of Early Awakeners but was drastically superior to that of Non-Awakeners (*p* < 0.001). These data demonstrate that approximately 13% of the cohort—representing one-third of all patients with good neurological recovery—followed this delayed recovery pattern, a phenotype that renders early static assessments largely ineffective.

### 3.4. Predictors of the Late Awakener Phenotype

Given the clinical challenge of distinguishing Late Awakeners from Non-Awakeners during the initial 48 h, we performed a multivariate logistic regression analysis to identify independent predictors of delayed recovery among patients with initial poor motor responses (Table 2). After adjusting for age, sex, and resuscitation factors, favorable pre-hospital factors—specifically a shockable initial rhythm—remained the sole robust predictor associated with the Late Awakener phenotype. Patients with a shockable rhythm had a nearly ninefold higher likelihood of being a Late Awakener compared to a Non-Awakener (Adjusted Odds Ratio [aOR] 8.67, 95% CI 3.93–19.11, *p* < 0.001). In contrast, other factors including witnessed arrest (*p* = 0.722), bystander CPR (*p* = 0.322), and time to ROSC (*p* = 0.259) did not show a statistically significant association in the multivariate model. This suggests that the Late Awakener phenotype is not merely a function of shorter ischemic time, but is intrinsically linked to the reversible pathophysiology characteristic of shockable rhythms, distinct from the irreversible injury seen in non-shockable cases.

## 4. Discussion

In this study of comatose OHCA survivors treated with TTM, we moved beyond conventional static prognostication by applying a cluster analysis to serial motor scores. The principal finding of our study is the identification of three distinct longitudinal phenotypes of consciousness recovery: Early Awakeners, Late Awakeners, and Non-Awakeners.

Most notably, we identified a clinically critical subgroup—the Late Awakeners—who comprised approximately 13% of the total cohort and one-third of all patients with favorable outcomes. These patients presented with profound coma (GCS-M ≤ 2) during the first 48 h, making them indistinguishable from patients with irreversible brain injury based on early clinical examination alone. However, they subsequently demonstrated a delayed surge in neurological recovery after 72 h and achieved a remarkably high rate of good neurological outcome (79.6%) at 6 months.

Our multivariate analysis further revealed that a favorable pre-hospital factor, specifically a shockable initial rhythm, was the sole robust independent predictor of this delayed recovery phenotype.

Current international guidelines, including those by the ERC/ESICM, recommend delaying prognostication until at least 72 h post-ROSC to minimize false-positive predictions of poor outcome [5]. However, our findings suggest that strict adherence to static snapshots within this window may be perilous for a significant minority [26].

In our Late Awakener cohort, the mean GCS-M score was indistinguishable from that of Non-Awakeners at 48 h (mean ≤ 2.0) before surging to 4.7 at 72 h. In a strict binary algorithm, a patient with a motor score of 1 or 2 at 48 h might be prematurely classified as having a poor prognosis, potentially triggering discussions about the withdrawal of life-sustaining therapy (WLST).

Our data challenges this static approach by demonstrating that the trajectory (slope) of recovery is as important as the absolute score. This aligns with recent findings by Lagebrant et al. who highlighted that diagnostic uncertainty remains high in a subset of patients and that early pessimism often leads to premature WLST [18,19].

Most critically, our multivariate analysis provides a concrete marker to navigate this uncertainty: the presence of a shockable initial rhythm. Since this was the sole independent predictor of the Late Awakener phenotype (aOR 8.67), a lack of early motor response in patients with a shockable rhythm should be interpreted with extreme caution—likely representing a temporary “neuronal stunning” rather than permanent injury—and warrants an extended observation window.

Several pathophysiological mechanisms may explain the phenomenon of delayed awakening. First, the clearance of sedatives is often prolonged in post-cardiac arrest patients due to therapeutic hypothermia-induced reduction in hepatic metabolism and renal clearance [12]. Although our protocol minimized neuromuscular blockade and utilized short-acting agents, individual variations in drug metabolism could still contribute to prolonged unresponsiveness.

Second, and perhaps more importantly, the hypothesis of “neuronal stunning” offers a compelling explanation. Our analysis revealed that Late Awakeners shared favorable resuscitation features—such as short anoxic times (median 2.0 min) and high rates of shockable rhythms (72.2%)—that were comparable to those of Early Awakeners, yet distinct from Non-Awakeners (median 5.0 min, 21.3%).

This suggests that the initial prolonged coma in Late Awakeners was likely not driven by severe structural injury (as seen in Non-Awakeners), but rather by reversible synaptic failure or metabolic depression. In these patients, the neural networks may require a longer period to re-establish connectivity, analogous to the “myocardial stunning” seen after ischemia-reperfusion. This hypothesis is strongly supported by our multivariate finding that a shockable rhythm was the sole independent predictor of this phenotype, implying that the underlying pathology was functional and reversible rather than structural and permanent.

Additionally, cerebral edema represents another potentially reversible mechanism contributing to delayed awakening. Post-ischemic cytotoxic and vasogenic edema can cause transient mass effect and elevated intracranial pressure, leading to depressed neurological function that may gradually resolve over days as edema subsides. This temporal pattern of edema formation (typically peaking at 3–5 days) and subsequent resolution aligns with the delayed recovery trajectory observed in our Late Awakener phenotype.

We acknowledge that Figure 1 reveals a subtle visual difference in early GCS-M scores between Non-Awakeners and the other phenotypes during the first 48 h, despite the lack of statistical significance. These small early differences, while not meeting statistical thresholds in our cohort, may represent an early signal of recovery potential that could become statistically detectable in larger validation studies. However, the clinical relevance of such marginal differences at the individual patient level remains uncertain, as a GCS-M difference of less than 1 point is unlikely to alter bedside decision-making. We therefore emphasize that the trajectory of change, rather than absolute early values, should guide prognostic interpretation. Future multi-center studies with larger sample sizes may help clarify whether these subtle early variations carry independent predictive value.

Our findings have critical implications for WLST decision-making. Specifically, they highlight the persistent risk of a ‘self-fulfilling prophecy’, where early pessimism drives the withdrawal of care, inadvertently ensuring the poor outcome it sought to predict [16,27].

Our multivariate analysis identified a shockable initial rhythm as the single most critical indicator for caution. In our model, this factor increased the odds of delayed awakening by nearly nine-fold (aOR 8.67; Table 2). Consequently, clinicians should exercise extreme caution before making early WLST decisions in patients presenting with a shockable rhythm, even if motor responses remain absent at 48–72 h.

Rather than interpreting a persistently low GCS-M score in these patients as definitive evidence of irreversible injury, it should be regarded as a signal to extend the observation window (e.g., to 5–7 days) and to mandate confirmation with multimodal prognostication tools before considering the limitation of care. This strategy aligns with a precision prognostication approach, mitigating the risk of premature withdrawal in patients with viable recovery potential.

Based on our findings, we propose a tentative modification to current prognostication practice for patients with shockable initial rhythms who remain unresponsive at 72 h: (1) Rather than interpreting persistent coma (GCS-M ≤ 2) at 72 h as evidence of poor prognosis, clinicians should consider this a potential “Late Awakener” phenotype requiring extended observation; (2) For such patients, we recommend extending the observation period to at least 5–7 days before initiating WLST discussions; (3) During this extended window, serial neurological examinations should be performed daily, with particular attention to any trend toward improvement; (4) Multimodal prognostication (including EEG, somatosensory evoked potentials, and neuroimaging) should be employed to confirm irreversibility before WLST. This approach aligns with the ERC/ESICM 2021 guidelines’ emphasis on multimodal assessment but extends the temporal framework for this high-potential subgroup.

This study has several limitations. First, as a retrospective single-center study, the results may lack external generalizability, although our TTM protocol aligns with international standards. Second, while we utilized short-acting sedatives and minimized neuromuscular blockade, we could not quantitatively adjust for cumulative sedative doses in the clustering model. However, the distinct association of the Late Awakener phenotype with shockable rhythms—rather than renal or hepatic dysfunction variables—suggests that prolonged sedation is unlikely to be the primary driver of delayed awakening in this cohort. Third, we focused primarily on serial GCS motor scores. While this provides a universally accessible metric, incorporating other modalities such as serial EEG patterns or quantitative biomarkers (e.g., NSE) into the trajectory model could yield even more precise phenotypes. Fourth, we selected K-means clustering for trajectory modeling due to its computational efficiency, interpretability, and established use in clinical phenotyping studies. While alternative approaches such as latent class growth analysis (LCGA) or group-based trajectory modeling (GBTM) offer probabilistic cluster assignments and may better capture individual-level uncertainty, K-means provides discrete, clinically actionable phenotypes that are readily applicable to bedside decision-making. Future studies employing probabilistic trajectory models may provide additional insights into the heterogeneity within each phenotype. Future multi-center studies validating our Late Awakener profile and its specific link to shockable rhythms are warranted to refine prognostication guidelines further.

## 5. Conclusions

Consciousness recovery after OHCA is a heterogeneous process following distinct longitudinal trajectories. We identified a Late Awakener phenotype—comprising 12.9% of the cohort—that is clinically indistinguishable from Non-Awakeners in the first 48 h but carries a favorable long-term prognosis. Our findings strongly imply that for patients with a shockable initial rhythm—the sole independent predictor of this delayed recovery pattern—the absence of early motor recovery should not be interpreted as a sign of permanent neurological injury. Consequently, premature withdrawal of life-sustaining therapy (WLST) must be avoided in this specific subgroup. Instead, these patients warrant an extended observation period of at least 5–7 days to allow for the potential emergence of delayed recovery. Our findings underscore that a trajectory-based, rather than static, approach to neuroprognostication is essential to prevent the premature loss of patients with viable recovery potential.

## Figures and Tables

**Table 1:** Baseline Characteristics and Clinical Outcomes of OHCA Survivors Stratified by Consciousness Recovery Phenotype.

	Total (*n* = 417)	Early Awakener (*n* = 86)	Late Awakener (*n* = 54)	Non-Awakener (*n* = 277)	*p*-Value
Demographics					
Age (years)	56.0 (44.7–69.0)	48.9 (39.0–58.0)	56.5 (47.4–66.8)	58.0 (45.8–72.0)	<0.001
Male sex	298 (71.5%)	67 (77.9%)	44 (81.5%)	187 (67.5%)	0.038
Comorbidities					
Hypertension	136 (32.6%)	20 (23.3%)	17 (31.5%)	99 (35.7%)	0.096
Diabetes mellitus	97 (23.3%)	9 (10.5%)	7 (13.0%)	81 (29.2%)	<0.001
Resuscitation Factors					
Witnessed arrest	280 (67.1%)	73 (84.9%)	40 (74.1%)	167 (60.3%)	<0.001
Bystander CPR	251 (60.2%)	59 (68.6%)	38 (70.4%)	154 (55.6%)	0.032
Shockable rhythm	162 (38.8%)	64 (74.4%)	39 (72.2%)	59 (21.3%)	<0.001
Cardiac etiology	247 (59.2%)	79 (91.9%)	45 (83.3%)	123 (44.4%)	<0.001
Time Intervals					
Anoxic time (min)	4.0 (0.0–10.0)	2.0 (0.0–5.0)	2.0 (0.0–5.5)	5.0 (0.0–11.0)	<0.001
Time to ROSC (min)	31.0 (18.0–42.8)	18.0 (12.0–30.0)	21.5 (10.0–36.2)	36.0 (26.0–48.5)	<0.001
Clinical Outcomes					
Survival to discharge	234 (56.1%)	85 (98.8%)	50 (92.6%)	99 (35.7%)	<0.001
Good CPC (1–2)	131 (31.4%)	83 (96.5%)	43 (79.6%)	5 (1.8%)	<0.001

**Table 2:** Independent Predictors of Late Awakener Phenotype Among Patients with Initial Poor Motor Responses (vs. Non-Awakeners).

Variable	Adjusted Odds Ratio (aOR)	95% Confidence Interval	*p*-Value
Age (per 1-year increase)	1.00	0.98–1.03	0.795
Male Sex	1.72	0.67–4.45	0.227
Witnessed Arrest	1.16	0.50–2.67	0.722
Bystander CPR	1.54	0.66–3.62	0.322
Shockable Rhythm	8.67	3.93–19.11	<0.001
Time to ROSC (per min)	1.00	1.00–1.00	0.259

## Data Availability

The data presented in this study are available on request from the corresponding author. The data are not publicly available due to legal restrictions.

## Abbreviations

The following abbreviations are used in this manuscript:

OHCA	Out-of-Hospital Cardiac Arrest
ROSC	Return of Spontaneous Circulation
TTM	Targeted Temperature Management
GCS	Glasgow Coma Scale
GCS-M	Glasgow Coma Scale Motor Score
CPC	Cerebral Performance Category
WLST	Withdrawal of Life-Sustaining Therapy
HIBI	Hypoxic-Ischemic Brain Injury
NSE	Neuron-Specific Enolase
DNAR	Do-Not-Attempt-Resuscitation
NMBAs	Neuromuscular Blocking Agents
ERC	European Resuscitation Council
ESICM	European Society of Intensive Care Medicine
aOR	Adjusted Odds Ratio
CI	Confidence Interval
IQR	Interquartile Range
LOCF	Last Observation Carried Forward
